# Supplementary figures and images for: Delimiting the genera of the Ficinia Clade (Cypereae, Cyperaceae) based on molecular phylogenetic data
Source: PeerJ. 2021 Jan 26;9:e10737. doi: 10.7717/peerj.10737 (PMC7845527; doi:10.7717/peerj.10737)

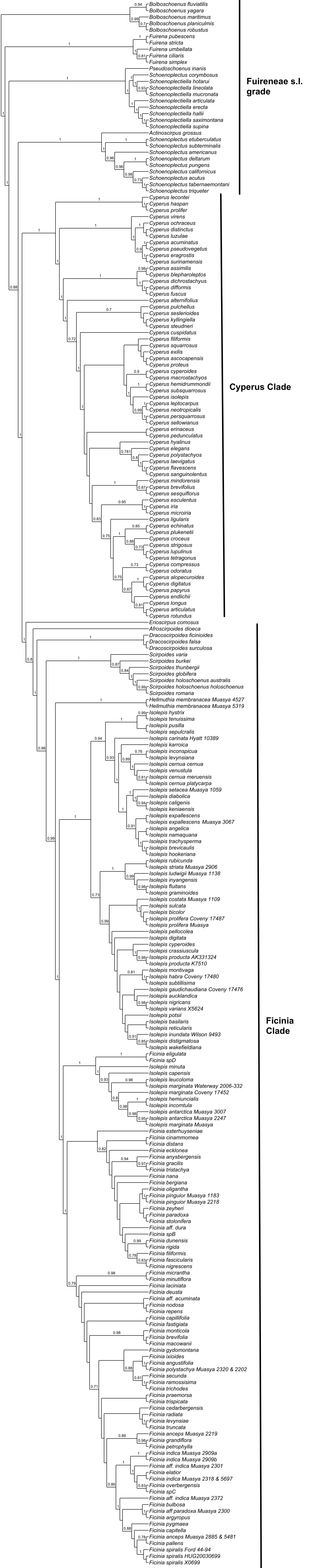

Supplement: Supplemental Information 5 [file peerj-09-10737-s005.pdf]

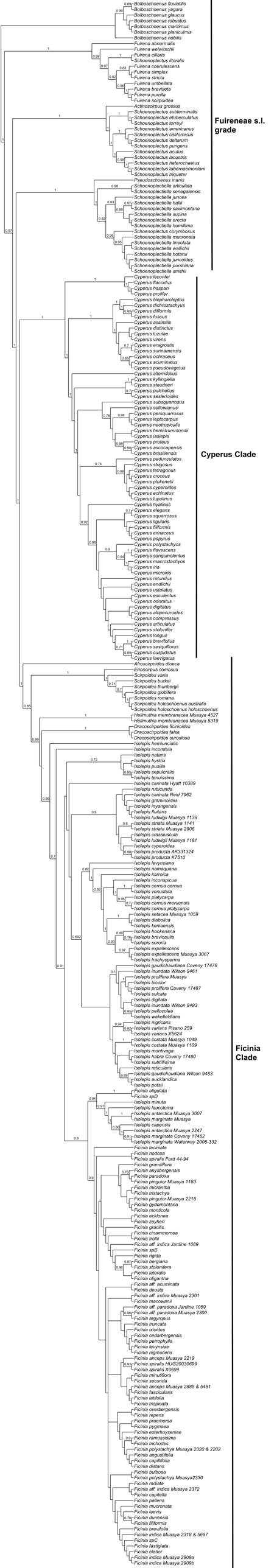

Supplement: Supplemental Information 6 [file peerj-09-10737-s006.pdf]

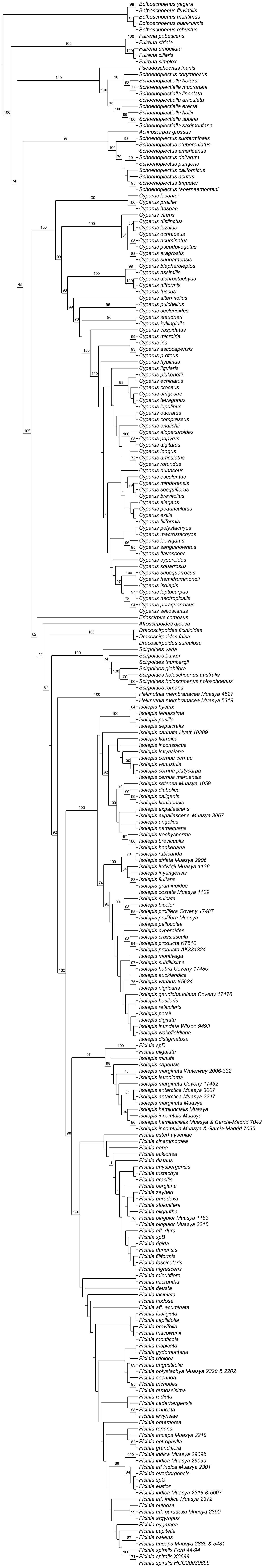

Supplement: Supplemental Information 7 [file peerj-09-10737-s007.pdf]

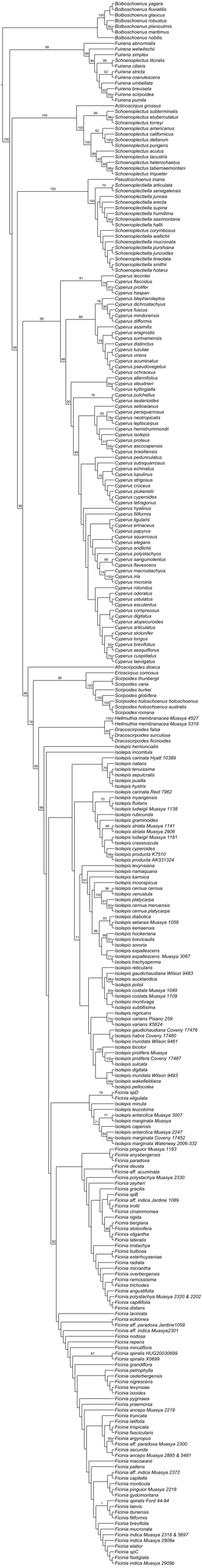

Supplement: Supplemental Information 8 [file peerj-09-10737-s008.pdf]
